# Predicting dihedral angle probability distributions for protein coil residues from primary sequence using neural networks

**DOI:** 10.1186/1471-2105-10-338

**Published:** 2009-10-16

**Authors:** Glennie Helles, Rasmus Fonseca

**Affiliations:** 1 University of Copenhagen, Department of Computer Science, Universitetsparken 1, 2100 Copenhagen, Denmark

## Abstract

**Background:**

Predicting the three-dimensional structure of a protein from its amino acid sequence is currently one of the most challenging problems in bioinformatics. The internal structure of helices and sheets is highly recurrent and help reduce the search space significantly. However, random coil segments make up nearly 40% of proteins and they do not have any apparent recurrent patterns, which complicates overall prediction accuracy of protein structure prediction methods. Luckily, previous work has indicated that coil segments are in fact not completely random in structure and flanking residues do seem to have a significant influence on the dihedral angles adopted by the individual amino acids in coil segments. In this work we attempt to predict a probability distribution of these dihedral angles based on the flanking residues. While attempts to predict dihedral angles of coil segments have been done previously, none have, to our knowledge, presented comparable results for the probability distribution of dihedral angles.

**Results:**

In this paper we develop an artificial neural network that uses an input-window of amino acids to predict a dihedral angle probability distribution for the middle residue in the input-window. The trained neural network shows a significant improvement (4-68%) in predicting the most probable bin (covering a 30° × 30° area of the dihedral angle space) for all amino acids in the data set compared to baseline statistics. An accuracy comparable to that of secondary structure prediction (≈ 80%) is achieved by observing the 20 bins with highest output values.

**Conclusion:**

Many different protein structure prediction methods exist and each uses different tools and auxiliary predictions to help determine the native structure. In this work the sequence is used to predict local context dependent dihedral angle propensities in coil-regions. This predicted distribution can potentially improve tertiary structure prediction methods that are based on sampling the backbone dihedral angles of individual amino acids. The predicted distribution may also help predict local structure fragments used in fragment assembly methods.

## Background

The primary sequence of a protein is believed to define the three-dimensional (tertiary) structure of the protein and many attempts at predicting the tertiary structure from primary sequence has been made (see for instance [1] for an overview of the CASP VIII experiment).

The main reasons that predicting protein structure from sequence alone is so difficult, is that the possible ways the amino acids can twist and turn with respect to each other are enormous. However, large parts of most proteins are arranged in secondary structures like helices and sheets, in which the dihedral angles of the amino acids lie within fairly limited areas as can be observed in Ramachandran plots [2-4]. Fortunately, predicting secondary structures can be done quite accurately [5-8], and since roughly 60% of amino acids in most proteins are arranged in these secondary structures [9], the number of possible amino acid conformations is dramatically decreased by this information. When attempting to predict the tertiary structure of proteins, the intermediate step of determining the secondary structure is thus typically performed.

It is important to note, though, that even if all helices and sheets in a protein have been predicted correctly, finding the complete tertiary structure is still a problem of daunting size. First of all, the dihedral angles of residues in secondary structures are still relatively flexible. Secondly, the dihedral angles of residues in coil segments are very flexible and they do not show any simple recurrent pattern like those in helices and sheets.

By inspecting the Ramachandran plot of large sets of proteins it is evident that although coil residues generally populate a much larger and more diverse area than helical and strand residues, certain dihedral angles are nearly never encountered. Steric overlap between atoms in the side chains of adjacent resides are believed to be responsible for this, indicating that flanking residues have a significant effect on the dihedral angles of a given residue, but exactly how big an effect remains unclear. Erman et al. [10] showed that, although the exact structure cannot be unequivocally determined by flanking residues, the structure is largely affected by these. On the other hand, Kabsch et al. [11] have shown that identical sequences of five residues in different proteins may still adopt different structures, which means that the exact dihedral angles of a residue cannot be determined strictly from the local environment.

Predicting the exact dihedral angle area of a coil residue based only on flanking residues thus appears to be infeasible, but we may still be able to predict the most probable dihedral angle areas. When residues are predicted as helix or strand residues, we are also provided with a most probable dihedral angle area. Using this information, de novo protein structure prediction methods allow us to direct the search to areas of the dihedral angle space where we are most likely to find the correct conformation.

A predicted probability distribution can therefore be used as either an alternative to fragment assembly, which, although it has improved tertiary structure prediction significantly, suffers from the fact that it relies heavily on known structures, or as a tool that can help improve the prediction success of the local fragment predictions used by fragment assembly algorithms [12-14]. A significant amount of work has already been done in predicting these local fragments [15-20], but as noted in [15], dihedral angle propensities are used in this prediction process and a neural network prediction of dihedral angle preferences could likely aide the prediction.

In this work we attempt to predict a dihedral angle probability distribution for coil regions that can be used by tertiary structure prediction algorithms to sample the conformational space more efficiently. Using a dihedral angle probability distribution does not restrict the dihedral angle space, but rather suggests a frequency to which we should search different areas of the dihedral angle space in order to increase the probability of finding the right dihedral angles for an amino acid.

Neural networks are well known for their ability to learn and extract patterns from massive amounts of data, so we have chosen to use this method to generate probability distributions. Neural networks have also previously played an important role in predicting secondary structures [5,7,8].

To our knowledge, predicting dihedral angle probability distributions of coil residues only have not previously been done. However, both Kuang et al. and Zimmermann and Hansmann have attempted to predict dihedral angle areas of coil residues and we have used them for inspiration. Both groups divide the Ramachandran plot into three main areas representing approximately 90-100% of the dihedral angle space and then they try to predict in which of the three areas the dihedral angles a coil residues would be in. Kuang et al. used both a neural network and support vector machine but they reported only marginal differences in performance for the two different prediction methods and ended up with an overall prediction accuracy of 77% for the 25% PDBSelect data set (February 2001 version) [21]. Zimmermann and Hansmann used support vector machines to create three classifiers; one for each part of the Ramachandran plot. They report a higher accuracy of between 81.7% and 93.3% for the 50% PDBSelect data set [22]. We wish to emphasize that unlike Kuang et al. and Zimmermann and Hansmann we are not concerned with predicting a single predefined area containing the correct dihedral angles. Instead, we attempt to predict a probability distribution that will yield the most probable dihedral angle area for a given residue in a given sequence. Hamelryck et al. developed a hidden markov model to predict probability distributions of dihedral angles [23], but their analysis was not limited to coil-regions and comparable results were not presented.

In the *Methods *section the method for constructing and training the neural network is described. Section *Discussion *presents and discusses the results, and section *Conclusions *draws the final conclusion.

## Results and Discussion

Two methods of evaluating the neural network are used. The first method measures the accuracy using only a single bin. The second include several bins and describe the accuracy of the predicted dihedral angle distribution.

### Lower bound on prediction accuracy

While a probability distribution can be constructed based on the results, the neural network is trained to predict a single bin. Table 1 shows the prediction accuracy for each type of amino acid. The prediction accuracy is the percentage of coil-residues for which the neural network had highest output in the bin corresponding to the correct dihedral angle. In order to determine the significance of the results presented, it is useful to compare them with the probability of guessing the right bin based on the distribution of dihedral angles in the data set. Simply guessing at the most populated bin for coil residues would yield a successful guess at a rate of:

**Table 1:** Improvements in prediction accuracy.

**AA-type**	**Property**	***G*^AA-type^**	**NN Prediction**	**Improvement**
arg	I	9.7%	13.6%	40.2%
asn	I	8.3%	13.3%	60.2%
asp	I	8.2%	13.7%	67.1%
gln	I	8.7%	13.3%	52.9%
glu	I	10.6%	15.1%	42.5%
his	I	7.7%	12.0%	55.8%
lys	I	9.7%	14.9%	53.6%
ser	I	10.9%	16.5%	51.4%
thr	I	9.1%	15.3%	68.1%
gly	-	15.0%	16.2%	8.0%
ala	O	12.4%	17.3%	39.5%
cys	O	10.5%	14.0%	33.3%
ile	O	14.3%	15.3%	7.0%
leu	O	12.5%	16.0%	28.0%
met	O	9.8%	12.3%	25.5%
phe	O	10.4%	12.6%	21.2%
pro	O	21.4%	27.0%	26.2%
trp	O	13.5%	15.2%	12.6%
tyr	O	9.4%	12.0%	27.7%
val	O	13.7%	14.2%	3.6%

Prediction accuracy of the neural network is compared to a lower bound derived from a purely statistical analysis of the data set. 'O' and 'I' in the "property" column denotes hydrophobic and hydrophilic residues respectively.

(1)

**Figure 1:**
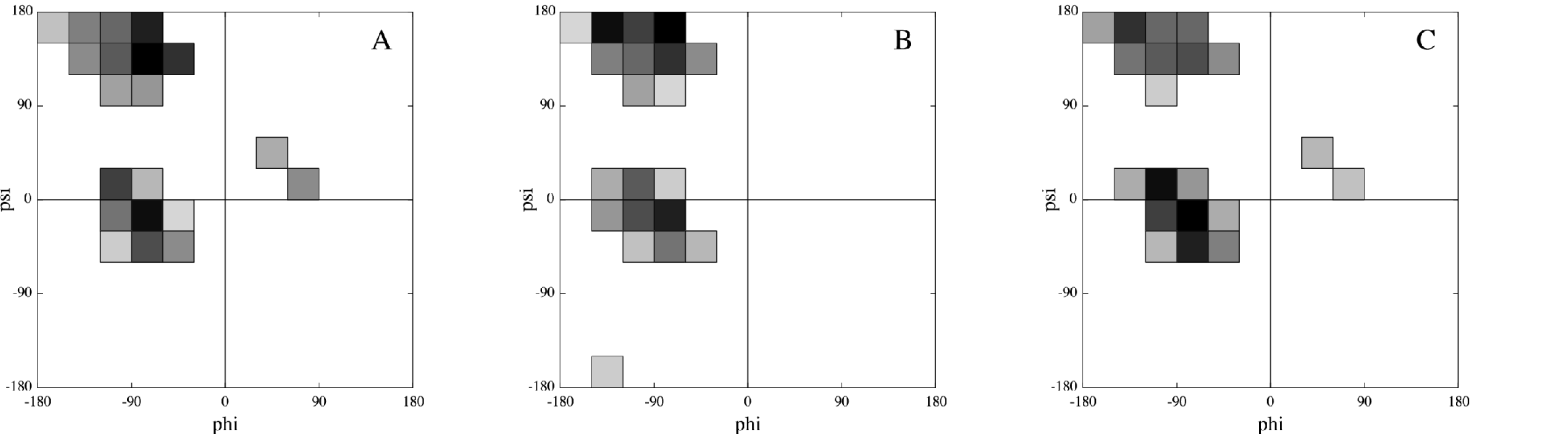
**Bin distribution**. The plot to the left (A) shows the distribution of the 20 most populated 30° × 30° bins for all coil residues in the training set. The plot in the middle (B) shows the distribution for just threonines in the training set, and the plot to the right (C) shows the predicted bins for threonine in the sequence Glu-Leu-Asp-Thr-Glu-Asp-Ala taken from a randomly chosen protein in the data set. The neighboring residues are used by the neural network to suggest a different distribution to yield a higher success rate. The darker the color of the bin the more likely it is that the angle set is within this bin.

Where *R*_most _is the number of residues in the most populated bin and *R*_total _is the total number of residues in the data set. We may think of *G *as a lower bound on the prediction accuracy. This lower bound can be tightened by analyzing plots specific to each type of amino acid. For instance Figure 1B shows the probability distribution for threonines that has been calculated using this equation. Lower bounds for the neural networks prediction accuracy, specific to each type of amino acid, *G*^AA-type^, can thus be determined.

As can be seen from Table 1 the trained neural network yield better accuracies than *G*^AA-type ^and the number of correctly predicted bins are improved for all types of amino acids. Improvements of more than 50% compared to guessing are observed for 7 out of the 20 residues. The largest improvement observed is for threonine where the correct bin is predicted by the neural network 68% more frequently than guessing at the most populated bin. Predicting dihedral angles for valine shows the smallest improvement of only 4%.

Interestingly, the neural network appears to perform better on hydrophilic residues, as 7 of the 9 hydrophilic residues are the ones that showed improvements of more than 50%. Only the hydrophilic residues, arginine and glutamic acid, showed improvements of less than 50% (but still >40%). In contrast, prediction for most hydrophobic residues showed improvements of less than 35%. This distinction between hydrophobic and hydrophilic residues may of course be mere coincidence, but it does seem to indicate that hydrophilic residues are much more controlled by their local environment than the hydrophobic residues, which are not as easily influenced. This is completely in keeping with the assumption that hydrophobic packing is the driving force in protein folding.

**Figure 2:**
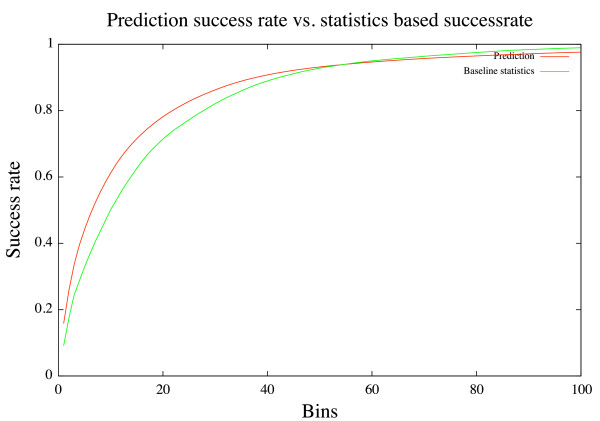
**NN prediction vs. baseline statistics**. Prediction vs. baseline statistics.

Guessing based only on the distributions observed in Ramachandran plots would yield a success rate of roughly 8-15% for all residues except proline that has an unusual high accuracy of 21%. Even large improvements of 4-68% will thus only bring the overall prediction accuracy up to roughly 12-27%, which is of course insufficient for reliable coil prediction. However, Figure 2 shows the prediction accuracy of the neural network compared to simple statistics based prediction when observing more than one bin. On average, neural network based prediction performs better as long as we look at an area that includes less than 55 bins. The highest gain in prediction accuracy compared to baseline statistics is achieved when we look at the 8 bins with highest output values.

### Accuracy of probability distribution

The above comparison with the lower bound indicates that the neural network is learning more than just baseline statistics, and that the flanking residues do in fact play a role for the local structure. However, our goal is not to predict a single bin, but rather to create a probability distribution for an area of the Ramachandran plot that will give us as high a prediction accuracy for any given sequence. With a prediction accuracy of ≈ 80% for secondary structures most tertiary structure prediction algorithms incorporates secondary structure predictions as a way to limit the search space. As already mentioned, residues in secondary structures do in fact span a rather large dihedral angle subspace, and so the question is whether we are able to obtain a similar accuracy for an equally sized area.

**Figure 3:**
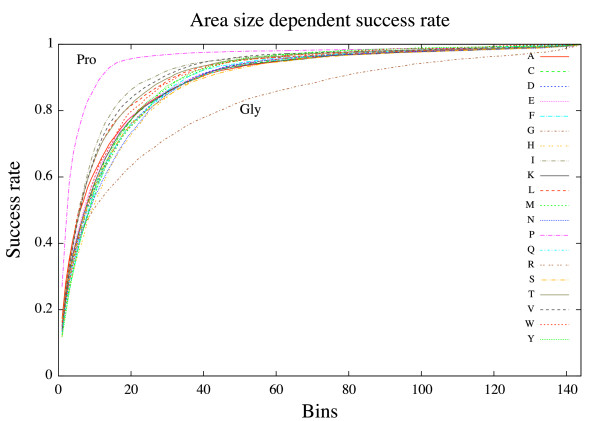
**Success rates**. Area size dependent success rate. Each bin represents a 30° × 30° area of the Ramachandran plot.

The increase in success rate for each included bin is depicted for each type of amino acid in Figure 3. As can be seen the average prediction accuracy for all residues is just under 80% (78%) within the 20 top scoring bins. For proline, which appears to be the easiest to predict, an accuracy of 80% is achieved within the dihedral angle area covered by the top eight scoring bins whereas glycine, which is by far the most difficult to predict, need to span an area covering 40 bins in order to achieve an ≈ 80% accuracy.

### Comparison

Both Kuang et al. [21] and Zimmermann & Hansmann [22], who attempted to predict dihedral angle areas of coil residues, divided the Ramachandran plot into three areas. Their smallest area (area A in [21], area H in [22]) has roughly the same size as 21 of our 30° × 30° bins. The second smallest area (area B in [21], area E in [22]) has an area corresponding to 25 of our bins and the largest area (referred to as area E/G in [21] and area O in [22]) corresponds to more than 80 of our bins - in fact in [22] area O simply takes up the remaining part of the Ramachandran plot.

Kuang et al. report an overall prediction accuracy of 77% and we thus achieve a higher accuracy per area ratio. Zimmermann et al. report an accuracy of 82.1% for area H, 81.7% accuracy for area E and 93.3% accuracy for their outlier area O. Again all areas are larger than ours and their improved accuracy over Kuang et al. are likely due to their use of the 50% PDBSelect data set, rather than the 25% PDBSelect data set used by both [21] and us. Generally, sequences with 50% or more sequence identity can be assumed to adopt the same three-dimensional structure whereas structures with only 25% cannot [24]. The classification algorithm used by Zimmermann and Hansmann thus have a natural advantage as their data set is not as diverse. Comparing the accuracy per area ratio, however, is not completely fair, since we are essentially trying to solve two different problems. Large areas like those in [21,22] are well suited for some tasks, but for limiting the search space in de novo protein structure prediction, we deem smaller bins more useful.

Figure 1C shows an example of the area predicted for threonine in a randomly chosen sequence from the data set. Figure 1A and 1B show plots drawn directly for all residues and only threonine in the data set respectively. The neural network clearly learns a different distribution based on the surrounding amino acids that will yield a better prediction accuracy for that specific sequence.

### Future work

An extension of the neural network, that may improve the results, would be to distribute the bins differently but still keep them relatively small. Preferred areas of turns [25] could be represented explicitly with bins or the optimal size of bins could be examined in more detail. Another possibility for future work is to assign higher target value to bins near the target (Φ, Ψ) point during training of the neural network. In this work the bin containing the target point is assigned 0.9 and all others 0.1. Due to the flexibility of the backbone the real point may easily be in one of the neighboring bins, so these could be assigned a target value of e.g. 0.5 during training. This could possibly help the neural network to generalize better.

Another extension is to train 20 individual neural networks, one for each amino acid. We have here chosen the network that had the best overall prediction accuracy for all of the amino acids, but from our experiments it is clear that individual residues often peaked at different times during the training procedure. We thus expect that the results we have reported here can be improved by training a network for each amino acid type.

## Conclusion

Our work shows that artificial neural networks can predict a probability distribution of dihedral angle areas for residues in a protein fast and better than simple statistics. For a dihedral angle area corresponding in size to those associated with helices and sheets that can be predicted with a ≈ 80% accuracy we achieve comparable results with a 78% accuracy. To our knowledge, results from attempts to predict probability distributions has not previously been reported, but it could prove very useful in guiding search algorithms for de novo protein structure prediction toward the most probable areas of the search space, much in the same way that predicted secondary structures do.

## Methods

A fully connected feed-forward neural network was constructed and used to predict a 30° × 30° dihedral angle bin corresponding to the (Φ, Ψ)-coordinates of the *target residue*.

We used the May 2008 25% PDBSelect data set , which consists of 3881 chains (553016 residues) with less than 25% sequence identity (20 chains were omitted in our data set because we were unable to obtain information about secondary structures with DSSP). In this experiment we are only interested in predicting probability distributions for coil residues, so we used information about secondary structures from the DSSP-algorithm [26]. A reduction from the eight groups of DSSP (3_10_-helix, *α*-helix, *π*-helix, *β*-bridges, *β*-sheets, turns and bends) was performed by classifying all residues that are either *β*-bridges, *β*-sheets, 3_10_-helices or *α*-helices as 'secondary structure' and the rest as 'coil'. This reduction corresponds to method A described in [27]. The neural network was trained on 'coil'-residues alone, though 'secondary structure' residues were often present in some part of the input window. Residues at the end of chains where either Φ or Ψ values are undefined were omitted.

The data set was split randomly in two equally sized sets, PDBSelect25_*A *_and PDBSelect25_*B*_. PDBSelect25_*A *_was used to determine an appropriate network configuration and PDBSelect25_*B *_was then used to obtain the prediction results reported in this work.

The input to the neural network was a window that spanned *W *residues of the amino-acid sequence with the target residue in the center. A number of experiments were run to determine the neural network configuration that would yield the highest prediction accuracy. Prediction accuracy was calculated as the percentage of coil residues from a validation set for which the neural network could predict the correct bin. Window sizes, *W*, of 5, 7 and 9 were used with various numbers of hidden neurons, *H*. Generally speaking, more hidden neurons are needed for larger input windows, but rather than experimenting with a fixed number of hidden neurons we simply kept increasing the number of hidden neurons with 50 until performance showed no improvements. Based on these experiments we settled on a window size of *W *= 7 and a neural network with *H *= 100 hidden neurons in a single hidden layer. We emphasize that while we have made experiments with many different architectures, we have not systematically verified that the neural network is optimal for this task, but as all architectures achieved almost the same prediction accuracy we feel confident that changing the architecture is unlikely to change the prediction accuracy in any major way.

**Figure 4:**
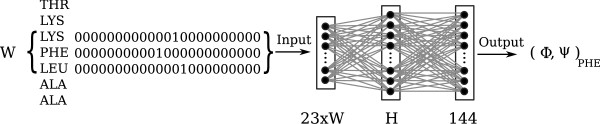
**Network configuration**. Workflow of the prediction. The residues in the input-window is encoded and used as input to the neural network that passes values through a hidden layer. The predicted (Φ, Ψ)-area can be read from the output-layer.

The neural network was designed so it had 23 input neurons per residue in the input window. One neuron was used to specify if the residue was part of a secondary structure (helix or strand), one was used to specify if the residue was part of a coil, one neuron was used to indicate if the input was a dummy (outside a chain or an unknown amino acid) and the 20 remaining input neurons were used to uniquely identify each of the 20 amino acid types. Neither the dummy nor the secondary structure input neurons are ever set to 1 for the middle residue. Using 20 input neurons to represent the residue is common and the procedure roughly corresponded to the one used by [7] to predict secondary structure. Figure 4 shows an overview of the neural network design.

The 144 neurons in the output-layer each correspond to a 30° × 30° area of the Ramachandran-plot. It was estimated that this size would be sufficiently small to be of use and sufficiently big to ensure that uncertainties in dihedral angles would not prevent the neural network from being able to learn. The expected output value of a certain area was 0.9 if the Φ and Ψ-angles of the middle residue of the input window fell within the boundaries of this bin, and 0.1 otherwise. We used 0.9 and 0.1 rather than 1 and 0 to ensure faster convergence with the standard logistic sigmoid activation function that was used in all layers. We used the standard sigmoid function because it is fast and because we are essentially only interested in finding the highest output signals and not the output value per se. The neural network was trained using standard back-propagation with learning momentum. The learning parameters of the back-propagation algorithm was set to *γ *= 0.05 (learning rate) and *α *= 0.1 (learning momentum).

For the initial experiments with different neural network configurations we split the PDBSelect25_*A *_data set randomly into five subsets. Four of them was used for training one for validation. Training was then carried out for 10.000 epochs with the weights updated after each training example. The highest prediction accuracy was achieved within the first 1000 epochs in all experiments. After 1000 epochs the prediction accuracy showed the slow decline for the unknown validation set and the slow increase in the training set that is the typical sign of over-fitting.

Once we settled on a neural network configuration we trained and validated the network on the PDBSelect25_*B *_data set. Like the PDBSelect25_*A *_data set, the PDBSelect25_*B *_data set split randomly into five subsets where four were used for training and one was used for validation. Since we previously achieved the highest prediction accuracy within the first 1000 epochs, we cut the training time down to 5000 epochs, but otherwise the hyper-parameters were identical to the ones already described. We ran a traditional 5-fold cross validation to ensure that the PDBSelect25_*B *_data set had not been split inappropriately. As is evident from Table 2, the neural network was able to predict the correct 30° × 30° bin approximately 16% of the times regardless of the way the data set was split.

**Table 2:** 5-fold cross validation results.

	**Prediction accuracy_*singlebin*_**
Split A	16.2%
Split B	15.0%
Split C	15.9%
Split D	15.9%
Split E	15.8%

Avg	15.7%

The data set was randomized and split into five separate sets and we carried out a 5-fold cross validation. The results from each fold is listed here along with the average.

## Authors' contributions

GH conceived of the study and carried out implementation of the neural network. RF has helped design the study and been responsible for data acquisition. Both authors have been involved in the literature study and both have drafted, read and approved the manuscript.

## References

[B1] Ben-David M, Noivirt-Brik O, Paz A, Prilusky J, Sussman JL, Levy Y (2009). Assessment of CASP8 structure predictions for template free targets.

[B2] Ramachandran GN, Ramakrishnan C, Sasisekharan V Stereochemistry of polypeptide chain configurations. J Mol Biol.

[B3] Barlow DJ, Thornton JM (1988). Helix geometry in proteins. J Mol Biol.

[B4] Sibanda BL, Blundell TL, Thornton JM (1989). Conformation of beta-hairpins in protein structures. A systematic classification with applications to modelling by homology, electron density fitting and protein engineering. J Mol Biol.

[B5] Jones D (1999). Protein secondary structure prediction based on position-specific scoring matrices. Journal of Molecular Biology.

[B6] Chu W, Ghahramani Z, Wild DL (2004). A graphical model for protein secondary structure prediction. ICML '04: Proceedings of the twenty-first international conference on Machine learning.

[B7] Qian N, Sejnowski TJ (1988). Predicting the secondary structure of globular proteins using neural network models. J Mol Biol.

[B8] Rost B, Sander C Prediction of Protein Secondary Structure at Better than 70% Accuracy. Journal of Molecular Biology.

[B9] Creighton T (1993). Proteins Structures and Molecular Properties.

[B10] Keskin O, Yuret D, Gursoy A, Turkay M, Erman B (2004). Relationships Between Amino Acid Sequence and Backbone Torsion Angle Preferences. Proteins: Structure, Function, and Bioinformatics.

[B11] Kabsch W, Sander C (1984). On the use of sequence homologies to predict protein structure: Identical pentapeptides can have completely different conformations. Proceedings of the National Academy of Sciences of the United States of America.

[B12] Klepeis JL, Floudas CA (2003). ASTRO-FOLD: A Combinatorial and Global Optimization Framework for Ab Initio Prediction of Three-Dimensional Structures of Proteins from the Amino Acid Sequence. Biophysical Journal.

[B13] Zhang Y, Wu S, Skolnick J Ab initio modeling of small proteins by iterative TASSER simulations. BMC Biology.

[B14] Martin Paluszewski PW (2008). Protein Decoy Generation Using Branch and Bound with Efficient Bounding. Algorithms in Bioinformatics.

[B15] Fernandez-Fuentes N, Oliva B, Fiser A (2006). A supersecondary structure library and search algorithm for modeling loops in protein structures. Nucl Acids Res.

[B16] Hunter CG, Subramaniam S (2003). Protein local structure prediction from sequence. Proteins: Structure, Function, and Genetics.

[B17] Fourrier L, Benros C, de Brevern AG (2004). Use of a structural alphabet for analysis of short loops connecting repetitive structures. BMC Bioinformatics.

[B18] Etchebest C, Benros C, Hazout S, Brevern AD (2005). A structural alphabet for local protein structures: improved prediction methods. Proteins: Structure, Function, and Bioinformatics.

[B19] Sander O, Sommer I, Lengauer T (2006). Local protein structure prediction using discriminative models. BMC Bioinformatics.

[B20] Katzman S, Barrett C, Thiltgen G, Karchin R, Karplus K (2008). Predict-2nd: a tool for generalized protein local structure prediction. Bioinformatics.

[B21] Kuang R, Leslie CS, Yang AS (2004). Protein backbone angle prediction with machine learning approaches. Bioinformatics.

[B22] Zimmermann O, Hansmann UHE (2006). Support vector machines for prediction of dihedral angle regions. Bioinformatics.

[B23] Boomsma W, Mardia KV, Taylor CC, Ferkinghoff-Borg J, Krogh A, Hamelryck T A generative, probabilistic model of local protein structure. Proceedings of the National Academy of Sciences.

[B24] Rost B (1999). Twilight zone of protein sequence alignment. Protein Engineering.

[B25] Perskie L, Street T, Rose G (2008). Structures, Basins and Energies: A Deconstruction of the Protein Coil Library. Protein Science.

[B26] Kabsch W, Sander C Dictionary of protein secondary structure: pattern recognition of hydrogen-bonded and geometrical features. Biopolymers.

[B27] Cuff J, Barton G (1999). Evaluation and improvement of multiple sequence methods for protein secondary structure prediction. Proteins: Structure, Function and Genetics.

